# Maxillary intraosseous hemangioma: case report

**DOI:** 10.11604/pamj.2024.48.135.43355

**Published:** 2024-07-25

**Authors:** Anas Douami, Oussama Marsafi, Hasnaa Belgadir, Aicha Merzem, Loubna Nashi, Omar Amriss, Nadia Moussali, Naima Elbenna

**Affiliations:** 1 Radiology Department, University Hospital of 20 August 1953, Faculty of Medicine and Pharmacy of Casablanca, Hassan II University, Casablanca, Morocco

**Keywords:** Intraosseous hemangioma, maxillary bone, CT scan, magnetic resonance imaging, case report

## Abstract

Maxillary intraosseous hemangiomas are rare benign vascular lesions, accounting for less than 1% of all primary bone tumors. Clinical examination often reveals a hard, painless swelling mass that is rarely pulsatile. Imaging not only helps to make a positive diagnosis but also contributes to therapeutic management. We report a case of a left maxillary intra osseous hemangioma Some authors have proposed exclusive embolization as a treatment for maxillary angiomas, but this requires several sessions for complete devascularization.

## Introduction

Intraosseous haemangiomas are rare benign vascular lesions. The most common sites are the spine and cranial bone. Maxillofacial haemangiomas are even rarer [1,2]. Its treatment is delicate, due to the high risk of intraoperative haemorrhage, hence the need to know its vascular nature preoperatively to be able to plan embolisation to minimise the risk of haemorrhage [3]. We report the case of a left maxillary intraosseous haemangioma, which was diagnosed based on pathognomonic features on cross-sectional imaging (CT-MRI).

## Patient and observation

**Patient information:** a 71-year-old female patient, presented a progressively enlarging mass on his left cheek evolving for the past three years; non-smoker, non-alcoholic, with no particular pathological antecedents.

**Clinical findings:** the mass was painless and caused no symptoms, apart from a hint of cosmetic deformity. On physical examination, a bony, hard, painless mass measuring approximately 1.5 × 1 cm was palpated on the lower left cheek. There were no other associated signs in the ipsilateral eye, and in particular no abnormalities of vision, eye movement or globe position; there was no regional paresthesia. The overlying skin was mobile and normal in appearance. No other specific signs were observed in the head or neck region. At the consultation, rhinoscopy was performed with 0° optics; after instillation of nasal vasoconstrictor, a complete left nasal obstruction was noted, preventing passage of the optic.

**Timeline of current episode:** September 2023: CT SCAN was performed. October 2023: Magnetic Resonance Imaging (MRI) was performed. November 2023: biopsy and histology and study were conducted. November 2023: patient referral to the healthcare unit.

**Figure 1:**
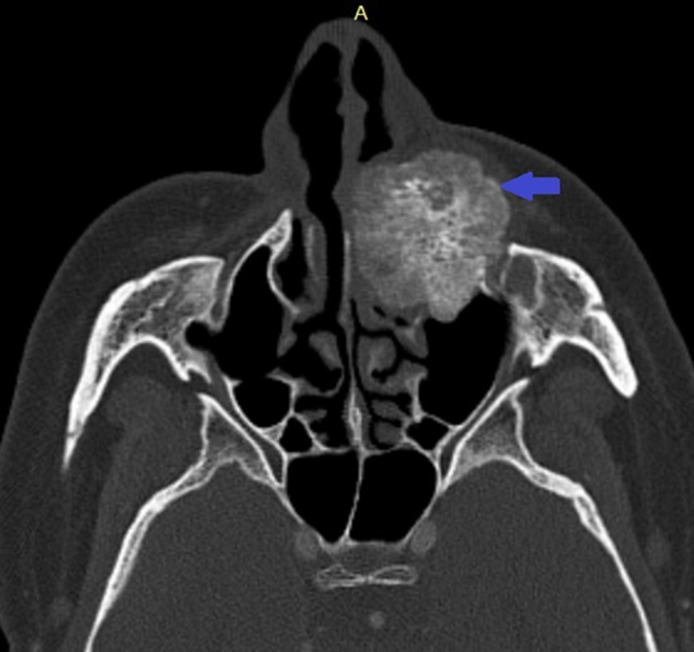
axial CT scans, viewed at wide window settings showing an intraosseous hemangioma arising from the left maxilla (blue arrow)

**Figure 2:**
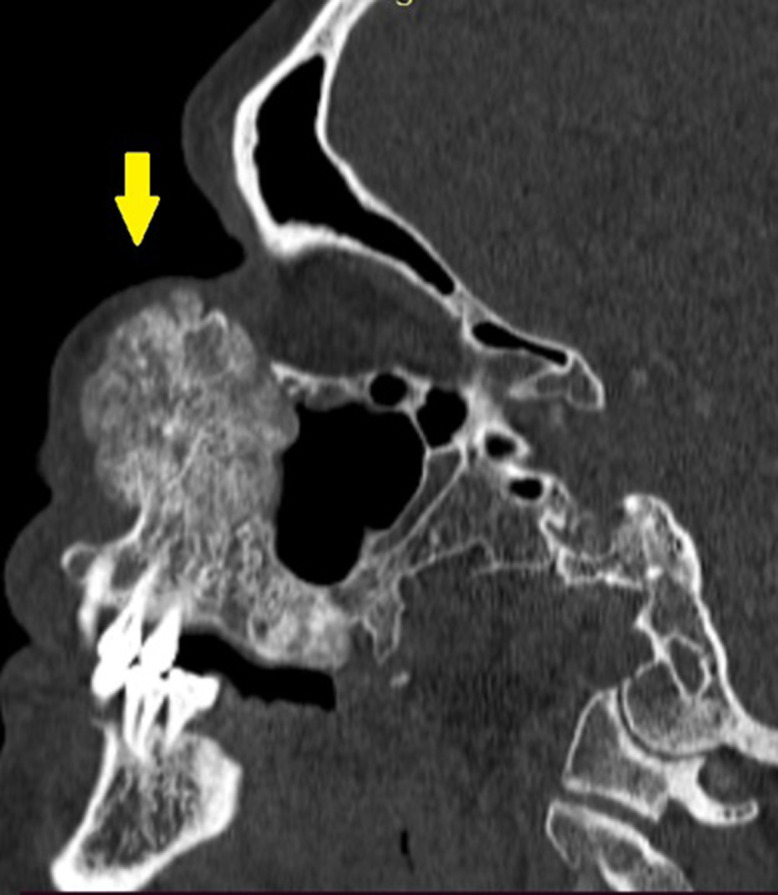
sagittal CT scans, viewed at wide window settings showing an intraosseous hemangioma arising from the left maxilla (yellow arrow)

**Figure 3:**
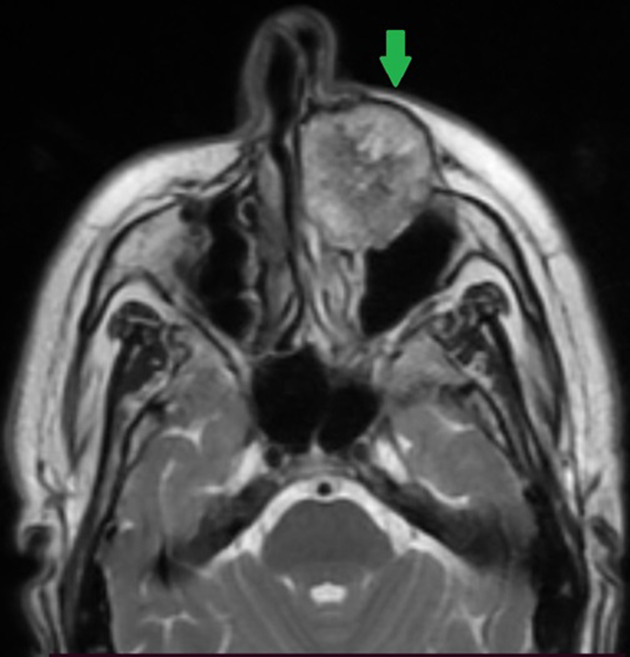
axial T2-weighted image showing a hyperintense lesion of the left maxilla (green arrow)

**Figure 4:**
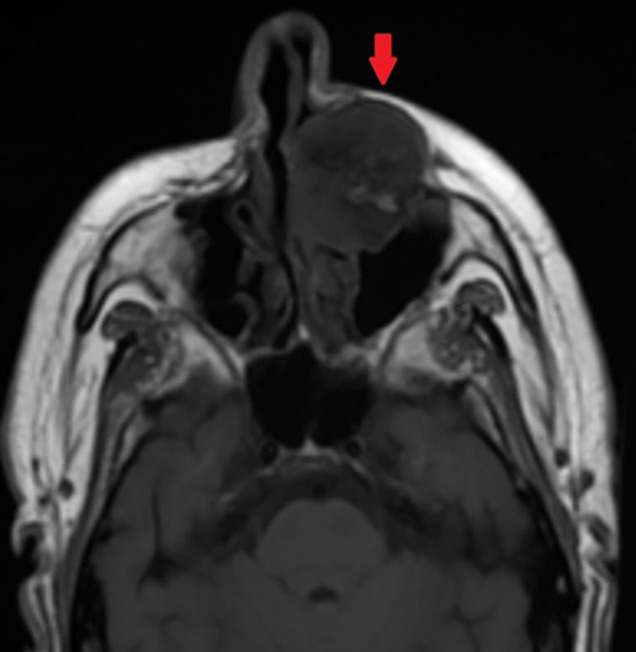
axial T1-weighted image showing a hyporintense lesion of the left maxilla (red arrow)

**Figure 5:**
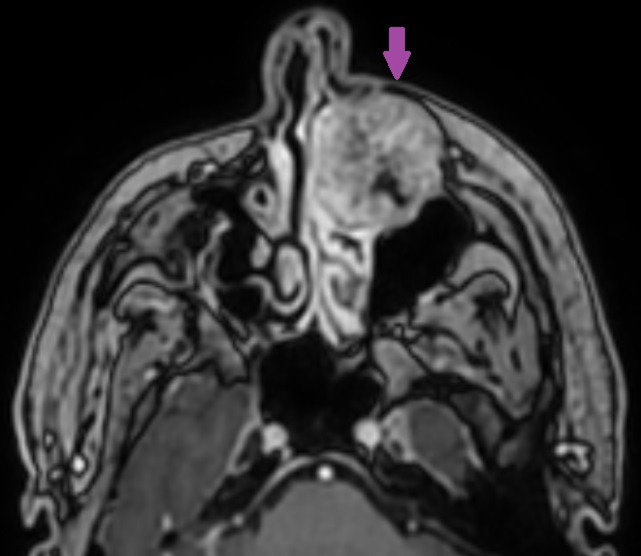
axial contrast-enhanced T1-weighted image showing marked enhancement of the left maxilla lesion (purple arrow)

**Figure 6:**
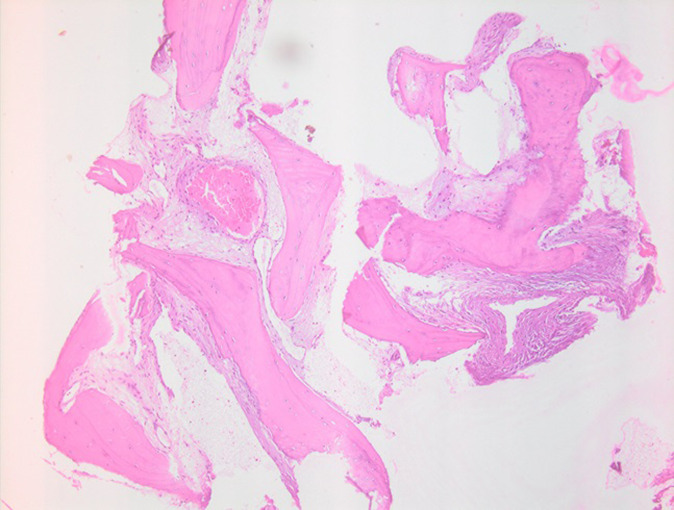
histological findings showing mature, lamellar trabecular bone, in which the intratrabecular space was completely occupied by large, dilated, thin-walled vessels, consistent with cavernous hemangioma of the bone

**Diagnostic assessment:** a contrast-enhanced facial CT scan showed a well-limited, rounded, expansive intraosseous bony lesion of the left maxillary bone (Figure 1, Figure 2). The process has a trabeculated, sunburst appearance, blowing away the bony cortices, which show very small interruptions in places, there was no periosteal reaction. MRI of the paranasal sinuses revealed a lesion of the left maxillary bone, which had an overall hypo to intermediate T1 signal intensity and a high T2 signal intensity with a marked enhancement after intravenous gadolinium injection (Figure 3, Figure 4, Figure 5). Within the mass were areas of signal void that corresponded to the trabeculae seen in the CT study. Intraorbital structures were intact, with no signs of infiltration, compression or displacement. The diagnosis was confirmed by a bone biopsy (Figure 6).

**Diagnosis:** the results were consistent with intraosseous cavernous hemangioma of the left maxillary bone.

**Therapeutic interventions:** surgical excision has been performed.

**Follow-up and outcome of interventions:** the patient had an excellent response he remains asymptomatic and in excellent clinical condition.

**Patient perspective:** “I have an expectation of being cured, given that some conditions have completely disappeared and I continue with clinical follow-up”.

**Informed consent:** the patient declares, in consequence of granting this permission, that he has no claim on the ground of breach of confidence or any other ground in any legal system against (author's/developer's name) and its agents, publishers, successors and assigns in respect of such use of the photograph(s) and textual material (case histories). The patient has given informed consent.

## Discussion

Intraosseous angioma is a benign bone tumor of undetermined etiology: true neoplasms or traumatic origin [4]. It accounts for 1% of symptomatic primary bone tumours [3]. Localization in the facial region is rare. It ranks third after vertebral and cranial sites [5]. Maxillary involvement is three times rarer than mandibular involvement [6]. Angiomas of the maxilla represent less than 1% of maxillary tumours [7]. Peak frequency usually occurs in the fourth and fifth decades of life [8]. literature reports a predominance of females, with a sex ratio of 2F/1H. They are classified histopathologically as cavernous or capillary type according to their vascular network. Almost all intraosseous hemangiomas of the facial skeleton are to be cavernous type [9].

The discovery of a bone lesion may be fortuitous during a routine radiological examination, but haemorrhage during dental avulsion is the most frequent circumstance for the discovery of maxillary angiomas. It can be cataclysmic or even lethal. It may also involve spontaneous, recurrent bleeding from the neck of a mobile tooth. Occasionally, the patient consults for facial asymmetry secondary to the hypoesthesia in a trigeminal territory. Clinical examination usually reveals a hard, painless swelling. Painless swelling, sometimes a pulsatile swelling with an audible murmur on auscultation [10].

CT scan is the most useful imaging technique because of its excellent characterization of trabecular and cortical detail and shows a well-circumscribed, rounded mass. It assesses extensions, relationships and mass effects in the vicinity of the tumor lesion [9,10]. The lesion has a “honeycomb” appearance, due to bone trabeculations [9]. When injected, the lesion takes on a strong contrast. MRI is the ideal modality to demonstrate mass-effect complications, such as neural impingement and extraosseous extension the signal intensity is somewhat variable, depending largely on the quantity of slow-moving venous blood and the amount of fat content on MRI, the lesion appears hypointense on T1-weighted images, hyperintense on T2-weighted images with intense enhancement after intraveinous gadolinium injection [8,10]. The differential diagnoses for intraosseous cavernous hemangioma include fibrous dysplasia, osteoma, Langerhans cell histiocytosis, dermoid tumor, and multiple myeloma.

Arteriography is specifically selected if a therapeutic procedure is envisaged, to check that there is no dangerous anastomosis between the two carotid axes. Arteriography reveals the feeding pedicles of the tumour and a vascular “blush” [8]. Some authors have proposed exclusive embolization as a treatment for maxillary angiomas, but it requires several sessions for complete devascularization [10]. However, the best treatment described to this day is surgery preceded by embolization to reduce the risk of bleeding. Embolization is not a risk-free procedure, and several complications have been described: vascular injury, hemiplegia, blindness, facial paralysis, catheter migration, thrombus formation, and mandibular avascular necrosis [10]. In the case of debilitating surgery, a second stage of reconstructive surgery must be planned. The evolution of this disease is very slow, and recurrences are possible after incomplete excesses.

## Conclusion

The maxillary bone localisation of hemangiomas is very rare. medical imaging plays a fundamental role in diagnostic and therapeutic management, often showing the very evocative, namely pathognomonic, appearance of intraosseous hemangioma.
